# Comparative analysis of ducks liver gene expressions infected with virulent or attenuated DHAV-3 reveals divergent host responses to viruses of different virulence

**DOI:** 10.3389/fmicb.2026.1740464

**Published:** 2026-02-04

**Authors:** Qiuling Fu, Wenlong Jiao, Chunhe Wan, Weiwei Wang, Jingliang Su, Xiaolong Liu, Longfei Cheng, Hongmei Chen, Jinhua Liu, Yu Huang, Guanghua Fu

**Affiliations:** 1 Institute of Animal Husbandry and Veterinary Medicine, Fujian Key Laboratory for Control and Prevention of Avian Diseases, Fujian Industry Technology Innovation Research Academy of Livestock and Poultry Diseases Prevention and Control, Fujian Academy of Agricultural Sciences, Fuzhou, China; 2 College of Veterinary Medicine of China Agricultural University, Beijing, China; 3 Present (Fuzhou) Biotech Co., Ltd., Fuzhou, China

**Keywords:** duck hepatitis A virus type 3, IFN-I, immunity, transcriptome, virulence

## Abstract

**Introduction:**

Duck hepatitis A virus type 3 (DHAV-3) causes acute fatal hepatitis in ducklings.

**Methods:**

This study compared host transcriptomic responses in duck livers following infection with virulent (HB) or attenuated (HB80) strains.

**Results:**

RNA sequencing (RNA-Seq) revealed that the virulent HB strain induced 2,355 differentially expressed genes (DEGs) at 2 days post-infection (dpi), compared to only 322 DEGs triggered by the attenuated HB80 strain. Functional analysis showed that the HB strain robustly activated immune pathways, particularly Toll-like receptor (TLR) and RIG-I-like receptor (RLR) signaling, leading to a potent type I interferon (IFN-I) response and marked chemokine upregulation. In contrast, the HB80 strain elicited a markedly milder immune reaction. Among the DEGs, 77 immune-related genes were identified, with significant enrichment in the IFN-I signaling pathway, suggesting their critical role in initiating an interferon storm and subsequent chemokine upregulation. Selected key genes (IFN-α2, RSAD2, RIG-I, MDA5, TBK1, TLR7) were validated by RT-qPCR and ELISA for targeted protein confirmation.

**Discussion:**

These findings delineate divergent host transcriptomic responses to virulent vs. attenuated DHAV-3 and highlight IFN-I signaling as a central axis in antiviral immunity.

## Introduction

Duck viral hepatitis (DVH) is an acute, highly fatal, and rapidly spreading viral infection of young ducklings less than 3 weeks old, characterized by hepatitis (Xu et al., 2012). The main causative agent, duck hepatitis A virus (DHAV), is classified as the sole member of the genus *Avihepatovirus* within the family *Picornaviridae* (Yugo et al., 2016). Its genome consists of a single positive-strand RNA molecule featuring a 5′-terminal methylated cap, three open reading frames, and a 3′-terminal poly(A) tail. DHAV has been classified into three serotypes: DHAV-1, the original and most widely distributed type; DHAV-2, reported thus far only in Taiwan and India (Tseng et al., 2007; Rajendran et al., 2023); and DHAV-3, an emerging type increasingly prevalent in mainland China, South Korea, Vietnam, and more recently in Egypt (Fu et al., 2024; Kim et al., 2007; Doan et al., 2016; Hassan et al., 2020; Yehia et al., 2021). Since the introduction of an officially approved DHAV-1 live vaccine in 2013, DHAV-3 has become the predominant pathogen responsible for DVH in China’s duck industry, resulting in substantial economic losses (Wen et al., 2018; Ou et al., 2022). To control this disease on duck farms, attenuated DHAV-3 vaccines, HB80, which is the first live attenuated DHAV-3 vaccine strain to be officially licensed in China, have been put into service against DHAV-3 since 2024 (Fu et al., 2024).

Research progress on *Avihepatovirus*, particularly regarding viral gene functions and virus-host interactions, undergoes a slow process. Although studies on gene structure and function have expanded rapidly following the initial genomic characterization of both highly virulent and attenuated DHAV-1 strains, most efforts have centered on DHAV-1(Song et al., 2014; Ou et al., 2017; Wang et al., 2024; Wang et al., 2025). Key genes of the highly virulent DHAV-1 strain including the capsid protein genes VP1 and VP3 (Li et al., 2022; Ou et al., 2017), and nonstructural protein genes 2A, 2B, 3C and 3D have been extensively studied (Wang et al., 2024; Liu et al., 2021; Zhang et al., 2025; Chen et al., 2018). In contrast, the gene functions and host interaction mechanisms of DHAV-3 remains largely explore.

RNA-Seq provides a systematic, probe-independent approach for analyzing transcriptional activity at single-nucleotide resolution, enabling comprehensive profiling of genome-wide expression patterns, alternative splicing events, and regulatory networks mediated by non-coding RNAs. Due to its probe-independent design and high quantification accuracy, RNA-Seq has become an essential tool for investigating pathogen-host interactions (Conesa et al., 2016). On DHAV-3 research, transcriptomic analyses of susceptible and resistant ducklings infected with the virulent strain have been used to identify differentially expressed genes relevant to genetic breeding (Zhang et al., 2018a,b; Cao et al., 2020). However, existing RNA-Seq studies have primarily focused on transcriptional changes induced by virulent DHAV-3 infection (Liang et al., 2021; Liang et al., 2022), comparative analyses between virulent and attenuated strains are still lacking.

In the present study, we aimed to fill this gap by infecting ducklings with two distinct DHAV-3 strains: the virulent HB strain and the attenuated HB80 vaccine strain. The HB strain was the highly hepatogenic virulent strain and its main target organ was the liver (Fu et al., 2024). The HB80 vaccine strain derived from HB is an attenuated strain that does not specifically target any particular tissue and is commonly used for preventing DHAV-3 in clinical settings. Using RNA-Seq, we comprehensively analyzed the transcriptomic profiles of duckling livers at 2 dpi with either strain. Selected DEGs were further validated using RT-qPCR, and corresponding protein expression levels were assessed by ELISA. Overall, this study provides the first comprehensive insight into global changes in hepatic gene expression following infection with either the highly virulent DHAV-3 strain or the attenuated vaccine strain.

## Materials and methods

### Virus and animals

The virulent DHAV-3 strain HB (GenBank number: PP713235) and attenuated live vaccine strain HB80 used in this study were obtained from College of Veterinary Medicine of China Agricultural University, with the viral titers of 10^6.17^ ELD_50_/mL and 10^5.17^ ELD_50_/mL, respectively. 150 one-day-old healthy Cherry Valley ducklings were obtained from Zhangzhou Changlong Agriculture and Animal Husbandry Co., Ltd. (Fujian, China). Throughout the entire experimental period, the ducklings were maintained in an isolation environment and had unrestricted access to food and water. All ducklings were healthy, negative for all major duck infectious pathogens, as confirmed via TaqMan real-time polymerase chain reaction (PCR) as previously described (Sun et al., 2019).

### Animal experimental design

A total of 150 three-day-old Cherry Valley ducklings were randomly divided into three groups and reared separately in three sterile isolators. Ducklings in the HB group received 0.5 mL of 10^5^ ELD_50_ of the virulent HB strain, while the HB80 group received 0.5 mL of 10^5^ ELD_50_ of the vaccine strain, both delivered by intramuscular injection into the thigh muscle. The control group were inoculated with 0.5 mL physiological saline. At 0.5, 2, 3, 7, and 14 dpi, five ducklings from each group were randomly selected, humanely euthanized by 100% compressed CO_2_ inhalation in a sealed chamber for 5 min, and a part of liver tissues were collected, snap-frozen in liquid nitrogen (for RNA detection) or fixed in 10% neutral-buffered formalin for histopathology. Formaln-fixed liver samples were processed routinely: 24 h fixation, paraffin embedding, 4 μm sections, hematoxylin & eosin (H&E) staining. Slides were examined blinded and were observed under the microscope.

### Library preparation for RNA-seq

Total RNA was extracted from 100 mg frozen liver sample using TRIzol^®^ Reagent according the manufacturer’s protocols. RNA integrity was checked using Agilent and quantified using NanoDrop ND-2000 spectrophotometer (Thermo Fisher Scientific Inc., MA, United States). Only samples with RQN ≥ 7 and 28S:18S ≥ 1.0 were used (Cao et al., 2020).

Subsequent steps (mRNA isolation, fragmentation, strand-specific library construction with Illumina^®^ Stranded mRNA Prep Ligation kit, 300–400 bp size selection, 10–15 cycles PCR, Qubit 4.0 quantification) were performed by Shanghai Majorbio Bio-pharm Biotechnology Co., Ltd. (Shanghai, China). Libraries were sequenced on NovaSeq X Plus (PE150) with the NovaSeq Reagent Kit (Illumina, CA, United States).

RNA purification, reverse transcription, library construction and sequencing were carried out at Shanghai Majorbio Bio-pharm Biotechnology Co., Ltd. (Shanghai, China), following the manufacturer’s protocols. The RNA-seq transcriptome library was prepared following Illumina® Stranded mRNA Prep, Ligation (San Diego, CA, United States), starting with 1 μg of total RNA. Briefly, messenger RNA was isolated via the polyA selection method by oligo(dT) beads and subsequently fragmented in a fragmentation buffer. Double-stranded cDNA was synthesized using random hexamer primers. The synthesized cDNA was then subjected to end-repair, phosphorylation, and adapter ligation following the library construction protocol. The resulting libraries were size-selected for cDNA fragments ranging from 300 to 400 bp using magnetic beads, and then amplified via PCR for 10–15 cycles. After quantification using a Qubit 4.0 fluorometer, the sequencing library was sequenced on a NovaSeq X Plus platform (PE150) with the NovaSeq Reagent Kit (Illumina, CA, United States).

### Quality control and read mapping

The genome of *Anas platyrhynchos* (GCF_000355885.1) was used as the reference sequence. Raw paired-end reads were trimmed and quality-controlled via fastp with default parameters (Chen et al., 2023). The resulting clean reads were then aligned to the reference genome in paired-end mode via HISAT2 (Kim et al., 2015). The mapped reads from each sample were assembled via StringTie via a reference-guided approach (Shumate et al., 2022).

### Differential expression analysis and functional enrichment

To identify DEGs across sample comparisons, gene expression levels were calculated via the reads per kilobase per million mapped reads (FPKM) method, with gene abundance quantified by RSEM (Sarantopoulou et al., 2021). Differential expression analysis was then conducted using DESeq2 (Love et al., 2014), with genes exhibiting |log₂FC| ≥ 1 and an adjusted FDR < 0.05 defined as significantly differentially expressed. Functional enrichment analyses followed, wherein Gene Ontology (GO) enrichment was assessed *via* Goatools and Kyoto Encyclopedia of Genes and Genomes (KEGG) pathway enrichment was analyzed using the SciPy library in Python, with both GO terms and KEGG pathways attaining *p*-values<0.05 deemed statistically significant. Subsequently, ClueGO was employed to analyze immune system processes associated with DEGs. Functional association networks of DEGs were constructed using the STRING database^1^, and protein–protein interaction (PPI) networks were visualized and analyzed through Cytoscape (version 3.9.1).

### RT-qPCR for confirmation

Total RNA was extracted from 50 mg frozen liver sample using the MagaBIO plus™ Total RNA Purification Kit (BioFlux, Hangzhou, China) according to the manufacturer’s protocols. RNA integrity and RNA concentration was performed as above. Each high-quality RNA was reverse-transcribed with the PrimeScript™ FAST RT Reagent Kit with gDNA Eraser (Takara, Dalian, China) following the manufacturer’s instructions. The obtained cDNA was stored at −20 °C until analysis.

To validate the RNA-seq results and assess mRNA expression levels of immune-related genes, 18 genes were validated with gene-specific primers (Supplementary Table S1) using TB Green^®^ Premix Ex Taq™ (Takara, Dalian, China) on an Achimed X4 system (Rocgene, Beijing, China). Extension temperature was primer-dependent (63–67 °C). RT-qPCR was carried out in accordance with what our previously established (Fu et al., 2016). Each sample was analyzed in triplicate, with β-actin as the endogenous reference gene.

### Enzyme-linked immunosorbent assay (ELISA)

To detect the protein expression levels of the key differential genes, liver homogenates (2 dpi) were assayed with duck-specific ELISA kits (Sumeike, Jiangsu, China) for RIG-I, TLR7, IFN-α2, CCL19 and RSAD2. Kits were manufacturer-validated for *Anas platyrhynchos*. Results are expressed as pg. mg^−1^ total protein. The procedures were carried out in accordance with the manufacturer’s instructions for the respective kits.

### Statistical analysis

The relative expression of DEGs in the HB, HB80 and control group was calculated with the 2^−∆∆Ct^ method, with the β-actin used as the reference gene. Gene expression was presented as the fold change relative to the control group. Data obtained in ELISA and RT-qPCR assays were expressed as mean ± standard deviation (SD). Group Differences were compared using two-way ANOVA in GraphPad Prism 10.4.1 (GraphPad Software Inc., CA, United States). *p*-value < 0.05 was considered statistically significant.

## Results

### Detection of viral infections in the liver

**Figure 1:**
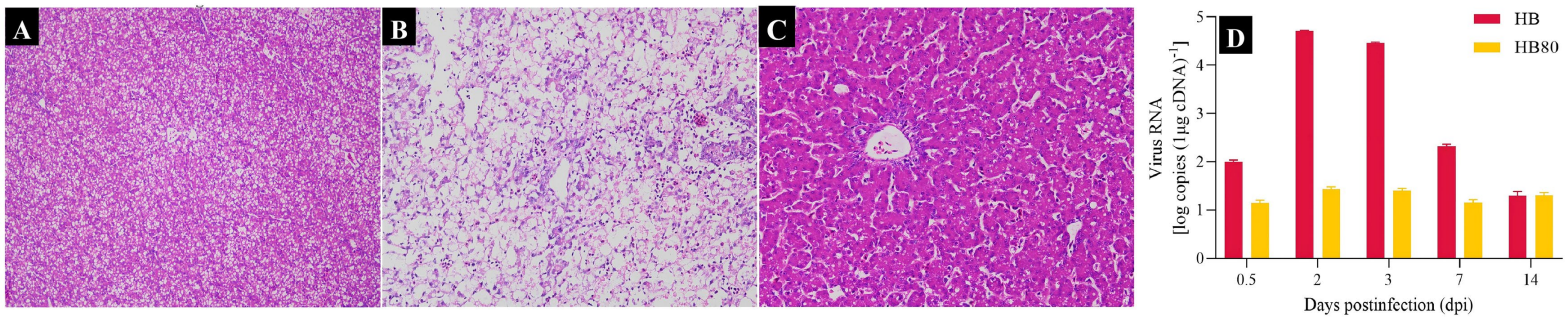
Pathological changes and viral load dynamics in the livers of HB-infected or HB80-immunized ducklings. **(A)** Normal liver histology in control ducklings. **(B)** Livers from HB-infected ducklings at 2 dpi exhibited widespread hepatocyte swelling and necrosis, accompanied by nuclear pyknosis and karyolysis. **(C)** No significant histopathological changes were observed in the livers of the HB80-immunized ducklings at 2 dpi. **(D)** Viral loads in the livers of ducklings with HB infection or HB80 immunization at 0.5, 2, 3, 7, and 14 dpi. The data are presented as mean ± standard deviation.

Anatomical analysis revealed that ducklings infected with the virulent DHAV-3 HB strain exhibited typical swelling and petechial hemorrhages across the liver at 2 dpi. In contrast, no visible lesions were observed in the livers of duckling in the HB80 group and the control group at the same point. Histological analysis revealed that ducklings in the HB group had widespread hepatic cells swelling and necrosis, along with nuclear pyknosis or karyolysis at 2 dpi (Figure 1A). However, no significant histopathological lesions were observed in the livers of the HB80 group (Figure 1B) or the control group during the same period (Figure 1C). We detected DHAV-3 loads in the livers using qPCR (Figure 1D). The results demonstrated that the virus was detectable at all time points in both the HB and HB80 groups. The HB group reached the highest viral load of 10^4.7^ copies/μg cDNA at 2 dpi, whereas the HB80 group exhibited a peak viral load of 10^1.38^ copies/μg cDNA at the same time point. Therefore, we selected the 2-dpi time point for RNA-seq analysis.

### DEGs after HB infection or HB80 immunization

To elucidate the differential mechanisms of liver injury induced by infection with virulent versus attenuated strains of DHAV-3, RNA sequencing libraries were prepared from 15 samples and sequenced on the NovaSeq™ X Plus platform in PE150 mode, generating a total of 748,731,728 raw reads (748.7 million). To ensure high-quality genome alignment and reliable differential gene expression analysis, the raw reads were filtered to remove low-quality sequences, resulting in 741,015,520 high-quality reads (741 million), accounting for 98.849% of the original data. These clean reads were then aligned to the mallard duck (*Anas platyrhynchos*) reference genome (GenBank: GCF_000355885.1). An average of 83.64% of the clean reads were uniquely mapped to the reference genome (Table 1), confirming the high integrity and reliability of the RNA-seq data for subsequent analyses.

**Table 1:** Number of reads of all bases detected via RNA-seq in HB-infected, HB80-immunized and control ducks.

Library	Number of raw reads	Number of clean reads	Number of uniquely mapped reads	Percentage of reads mapped (%)
HB-1	54,340,124	53,724,262	44,570,381	82.96
HB-2	56,347,744	55,734,142	45,961,001	82.46
HB-3	47,272,654	46,758,224	39,020,991	83.45
HB-4	54,374,146	53,750,416	44,800,791	83.35
HB-5	52,300,876	51,703,570	43,035,138	83.23
HB80-1	54,677,606	54,081,838	45,808,223	84.70
HB80-2	53,299,094	52,735,764	44,975,656	85.28
HB80-3	44,557,614	43,999,884	37,169,174	84.48
HB80-4	42,468,856	41,912,970	35,356,807	84.36
HB80-5	44,271,096	43,714,418	36,633,256	83.80
Control-1	44,471,954	43,974,292	36,921,633	83.96
Control-2	49,262,562	48,749,714	41,312,681	84.74
Control-3	50,161,726	49,537,148	40,609,442	81.98
Control-4	55,152,132	54,498,488	45,142,811	82.83
Control-5	45,773,544	45,240,390	37,525,923	82.95
Total	748,731,728	740,115,520	618,843,908	–

**Figure 2:**
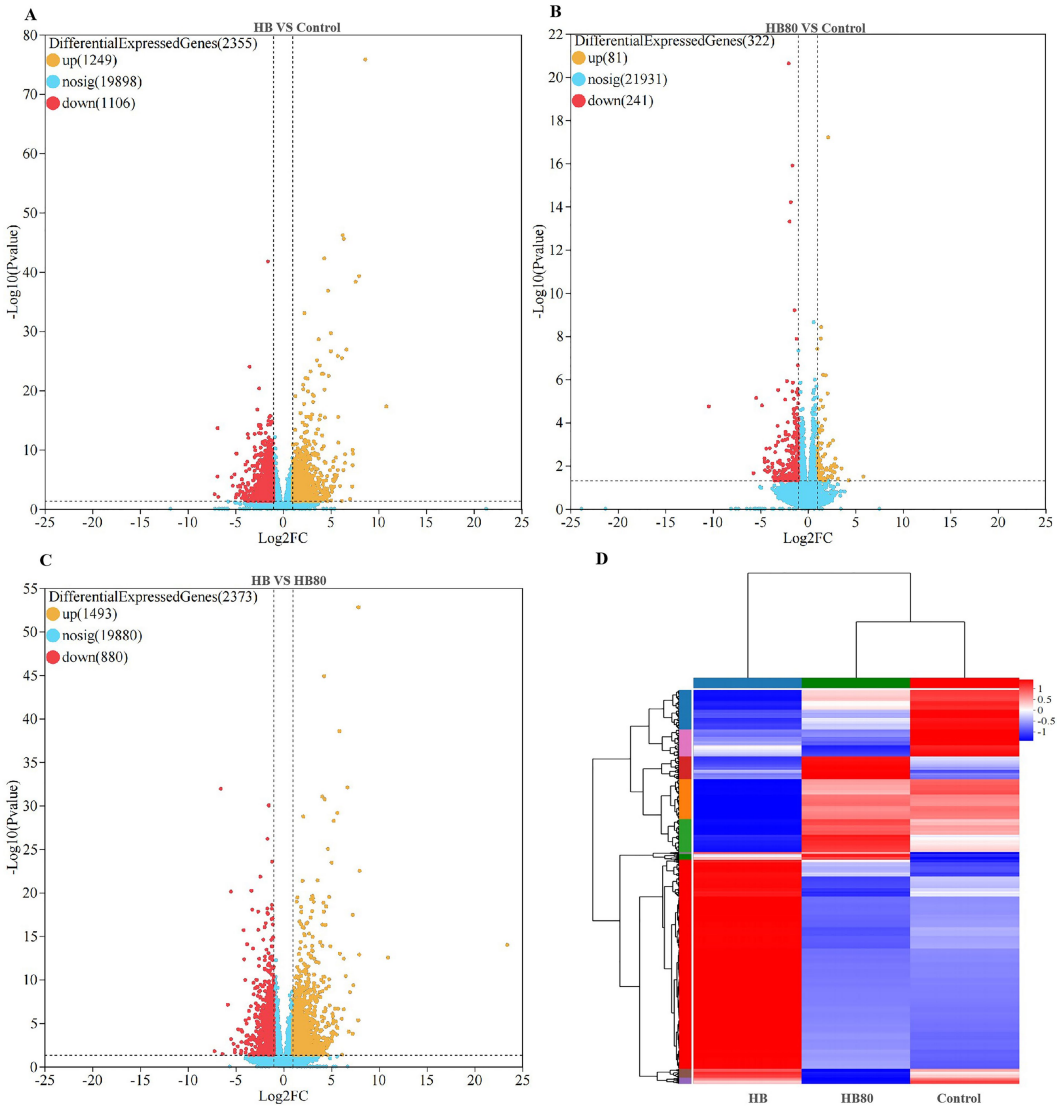
Quantitative analysis of DEGs. **(A–C)** Volcano plots depicting the distribution of DEGs. The *x*-axis represents the log2 fold change value, the *y*-axis represents -log_10_(*p*-value). Up-regulated, down-regulated, and non-significant genes are highlighted in yellow, red, and blue points, respectively. **(D)** Cluster heatmap of DEGs. The *x*-axis and *y*-axis represent the sample names and the normalized FPKM values (*Z*-score) of DEGs, respectively. Color intensity indicates expression levels, with red and blue representing high and low expression, respectively.

To screen DEGs in liver tissue after HB infection and HB80 immunization, the gene count values from each sample was analyzed using the DESeq2 software. Transcriptomic profiling revealed 2,355 DEGs in HB-infected ducks relative to the control ducks, including 1,249 upregulated and 1,106 downregulated genes (Figure 2A and Supplementary Table S2). In comparison, ducks immunized with the attenuated HB80 strain showed 322 DEGs relative to the control ducks, with 81 upregulated and 241 downregulated genes (Figure 2B and Supplementary Table S3). Importantly, a direct comparison between HB and HB80 groups identified 2,373 DEGs, of which 1,493 were upregulated and 880 were downregulated (Figure 2C and Supplementary Table S4). Hierarchical clustering was conducted on gene expression profiles based on FPKM values normalized by *Z*-score transformation. Distinct gene expression patterns were observed among HB-infected, HB80-immunized, and control groups, as illustrated in the cluster heatmap (Figure 2D). These findings demonstrated that both infection with virulent DHAV-3 and immunization with its attenuated vaccine induce extensive transcriptional reprogramming in the duck liver transcriptome.

### GO enrichment analysis of DEGs

**Figure 3:**
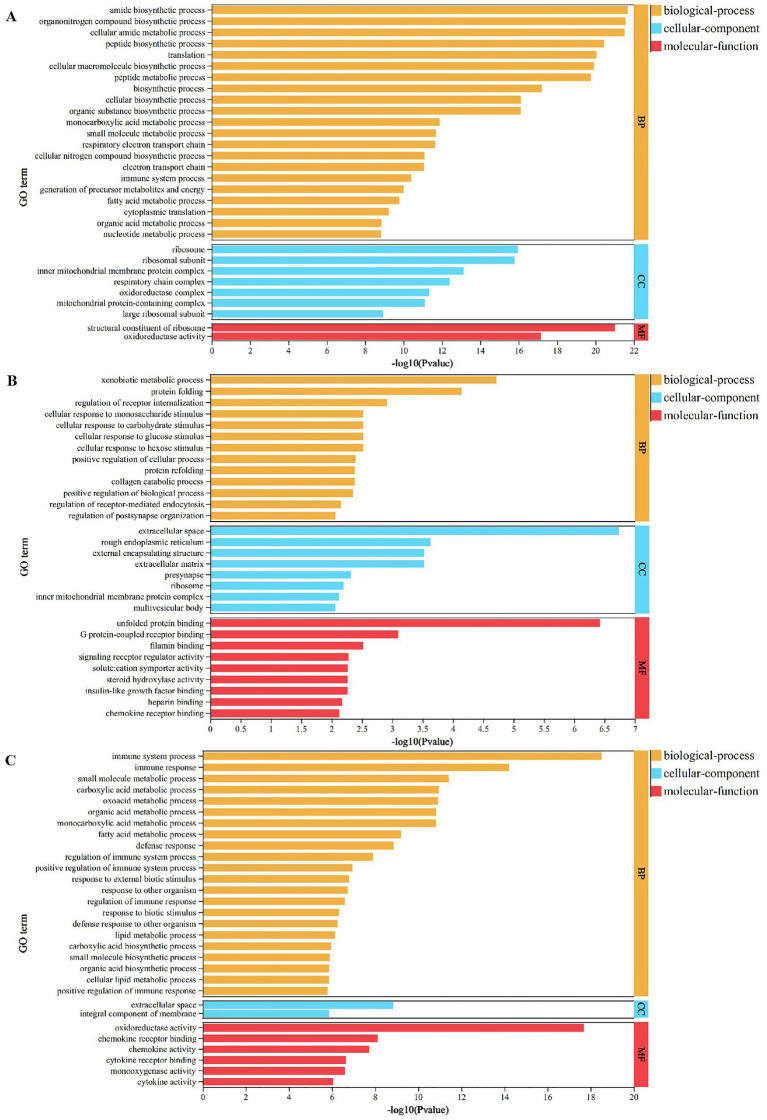
GO enrichment analysis of DEGs. GO terms were categorized into three domains: biological process (BP), cellular component (CC), and molecular function (MF). The top 30 significantly enriched GO terms (*p* < 0.05) are presented. **(A)** DEGs between the HB-infected and control groups. **(B)** DEGs between the HB80-immunized and control groups. **(C)** DEGs between the HB-infected and HB80-immunized groups.

To explore the biological functions associated with DEGs, GO enrichment analysis was conducted on DEGs identified from three comparisons: HB vs. control groups, HB80 vs. control groups, and HB vs. HB80 groups. The complete results were provided in Supplementary Tables S5–S7. As shown in Table 2, DEGs from the comparison of HB-infected and control ducks were annotated to a total of 477 GO terms, including 304 BP terms, 87 MF terms, and 51 CC terms. In contrast, DEGs from the HB80 vs. control groups were mapped to 356 GO terms, comprising 235 BP terms, 87 MF terms, and 34 CC terms. Additionally, DEGs identified from the comparison between HB and HB80 groups were associated with 498 GO terms, including 320 BP terms, 152 MF terms, and 26 CC terms. A bar chart depicting the top 30 significantly enriched GO terms was subsequently generated (Figure 3). Among the most significantly enriched GO terms in the HB-infected vs. control groups comparison were amide biosynthetic process (GO: 0043604), organonitrogen compound biosynthetic process (GO: 1901566), and cellular amide metabolic process (GO: 0043603) (Figure 3A). For the HB80-immunized versus control groups comparison, the top three enriched GO terms were extracellular space (GO: 0005615), unfolded protein binding (GO:0051082), and xenobiotic metabolic process (GO:0006805), as presented in Figure 3B. Notably, when comparing HB-infected and HB80-immunized groups, the most significantly enriched GO terms included immune system process (GO: 0002376), oxidoreductase activity (GO: 0016491), and immune response (GO: 0006955) (Figure 3C).

**Table 2:** A summary of GO terms after HB infection and HB80 immunization.

Groups	Numbers of BP	Numbers of MF	Numbers of CC	Total
HB vs. Control	304	122	51	477
HB80 vs. Control	235	87	34	356
HB vs. HB80	320	152	26	498

### KEGG pathway enrichment analysis of DEGs

**Figure 4:**
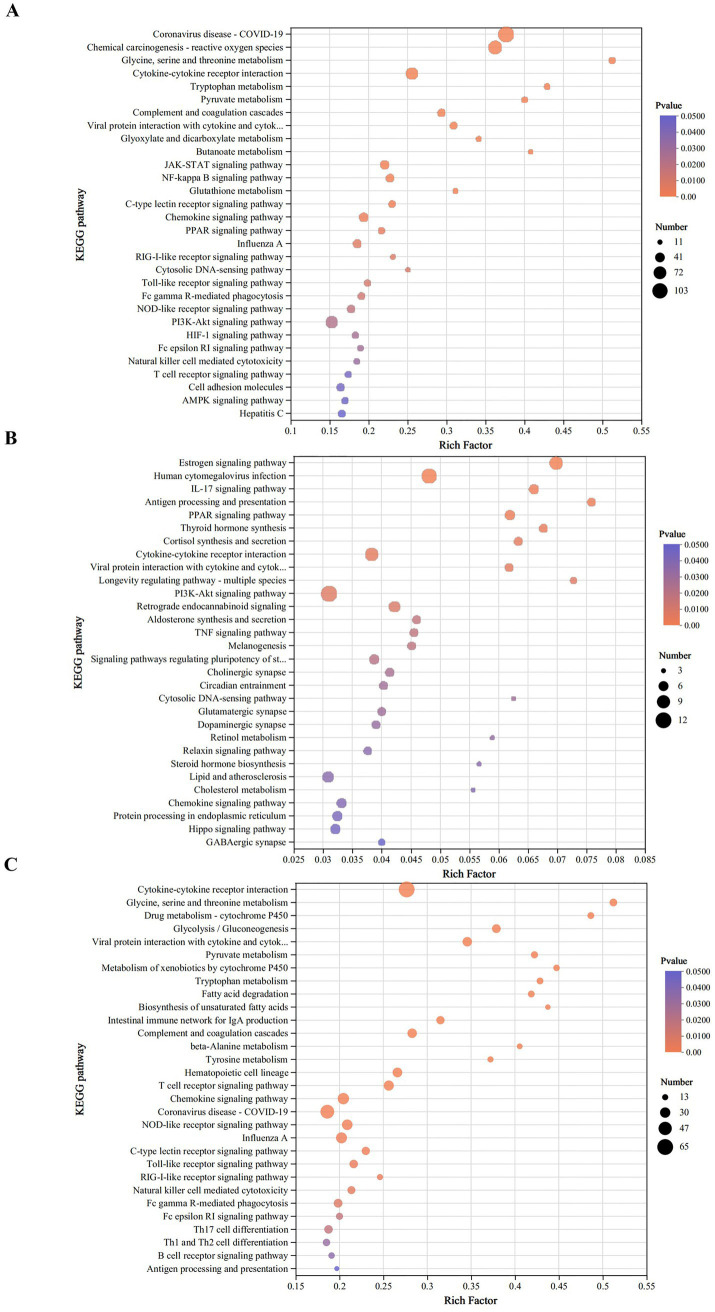
KEGG enrichment analysis of DEGs. Significantly enriched pathways are shown for three comparisons: **(A)** HB-infected vs. control, **(B)** HB80-immunized vs. control, and **(C)** HB-infected vs. HB80-immunized groups.

To elucidate the functional divergence between virulent and attenuated DHAV-3 strains, KEGG pathway enrichment analysis was performed on DEGs in duck livers following infection with either the virulent HB strain or attenuated HB80 vaccine strain of DHAV-3. Employing a significance threshold of *p*-value < 0.05, results were detailed in Supplementary Tables S8–S10. A total of 30 significantly enriched pathways were identified and are visually represented in the bubble plot shown in Figure 4.

In the comparison of the HB group vs. the control group, significant pathway enrichments were associated with metabolic processes, signaling molecule interactions, and immune responses-particularly those related to signal transduction. Key enriched pathways included energy metabolism, amino acid metabolism, cytokine-cytokine receptor interaction, TLR signaling pathway, RLR signaling pathway, and JAK–STAT signaling pathway (Figure 4A).

For the HB80 group compared to the control group, DEGs were predominantly enriched in pathways involved in the endocrine system, signaling molecule interactions, and immune responses. Notable pathways included the estrogen signaling pathway, IL-17 signaling pathway, antigen processing and presentation, cytokine-cytokine receptor interaction, and PI3K-Akt signaling pathway (Figure 4B).

Direct comparison between the HB and HB80 groups revealed enrichment of DEGs primarily in pathways related to signaling molecule interactions, immune responses, and metabolic processes. These encompassed cytokine-cytokine receptor interaction, amino acid metabolism, carbohydrate metabolism, TLR signaling, RLR signaling, and JAK–STAT signaling pathways. These results highlighted distinct molecular mechanisms underlying pathogenic infection compared to vaccine-induced immune responses (Figure 4C).

### Analysis of immune-related DEGs

**Figure 5:**
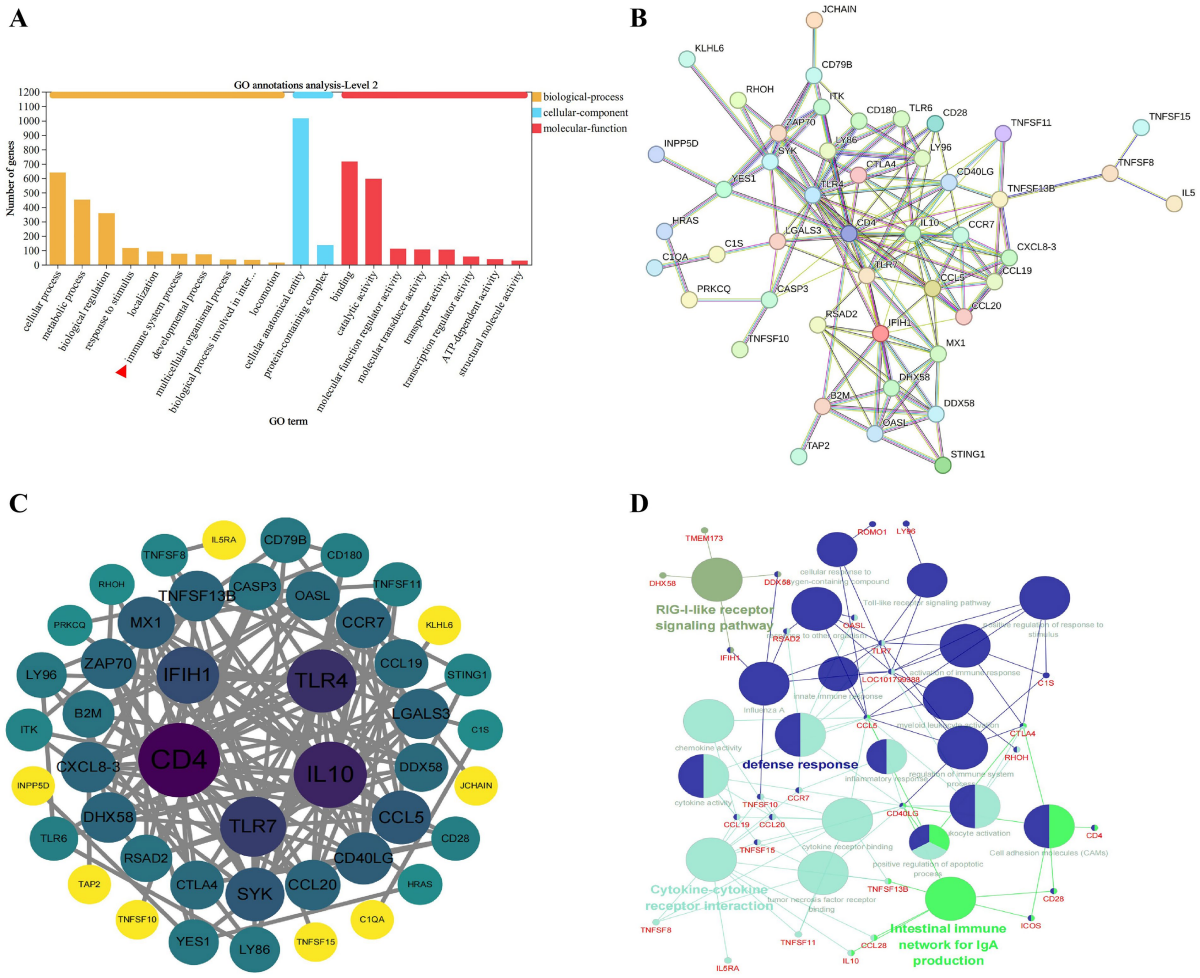
Analysis of immune-related DEGs. **(A)** GO annotation of DEGs from the HB-infected and HB80-immunized groups, categorized into biological process (BP), cellular component (CC), and molecular function (MF). The red triangle highlights the “immune system process” term. **(B)** STRING PPI network (55 nodes, 142 edges) highlights CD4, IL10, TLR4, TLR7, IFIH1, RSAD2, DHX58 as hub genes. **(C)** Cytoscape degree analysis confirms TLR7, IFIH1 and RSAD2 as top-ranking hubs. **(D)** ClueGO functional clusters show significant enrichment of RLR signaling, TLR signaling and cytokine–cytokine receptor interaction. Nodes represent significant GO or pathway terms (*p* < 0.05), edges represent term associations, and colors denote functional clusters.

Based on KEGG enrichment analysis, the virulent HB strain exhibited significant enrichment in immune-related signaling pathways compared to the attenuated HB80 strain. To further explore differences in immune-related gene expression between the two strains, we performed GO annotation analysis on DEGs (Supplementary Table S11). This analysis identified 77 DEGs associated with the immune system process (Figure 5A). After removing unannotated and redundant entries, 71 DEGs were selected for PPI network analysis to investigate functional correlations among these genes.

The 71 DEGs were submitted to the STRING database, resulting in a PPI network with 55 annotated nodes and 142 edges after omitting unconnected nodes (Figure 5B). Further analysis using Cytoscape identified several hub proteins, including CD4, IL-10, TLR4, TLR7, IFIH1, RSAD2, and DHX58, suggesting their potential key roles in mediating the differential host responses to DHAV-3 virulent vs. attenuated strains (Figure 5C). Functional enrichment analysis using ClueGO visually illustrated the connectivity and biological roles of these DEGs (Figure 5D), highlighting involvement in the RLR signaling pathway, TLR signaling pathway, and cytokine-cytokine receptor interaction.

### Confirmation of DEPs by RT-qPCR

**Figure 6:**
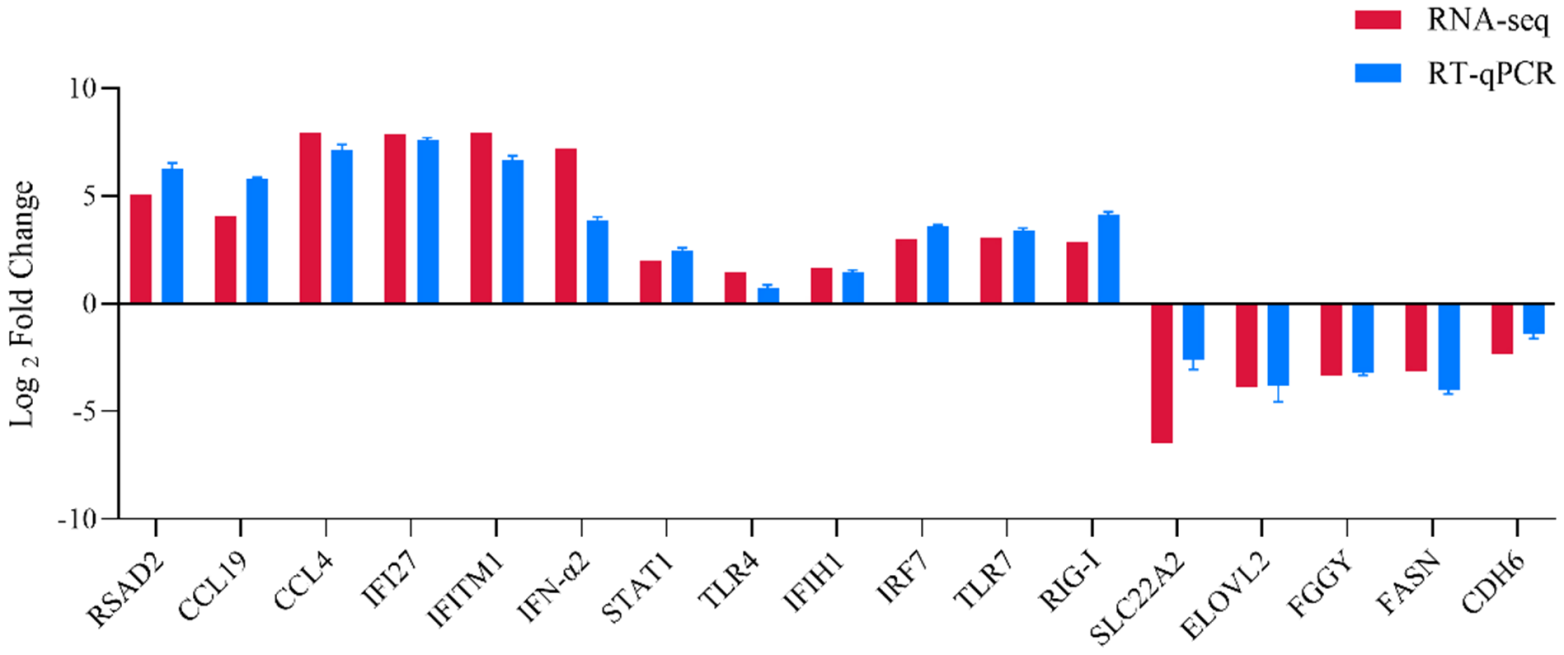
Correlation analysis between RNA-seq and RT-qPCR. Expression levels of selected genes were validated by RT-qPCR and compared with the RNA-seq data. The red and blue bars represent the normalized expression values from RNA-seq and RT-qPCR, respectively.

To further validate the expression patterns of DEGs identified via transcriptome sequencing, we selected 17 DEGs involved in innate immunity and antiviral response for RT-qPCR analysis. The qPCR results demonstrated that the expression changes of these genes, including *Rsad2, CCL19, CCL4, IFI27, IFITM1, STAT1, TLR4, IFIH1, IRF7, TLR7, DDX58, IFN-α2, SLC22A2, ELOVL2, FGGY, FASN* and *CDH6*, were consistent with the results of transcriptome sequencing (Figure 6). These findings confirmed that the gene expression trends observed via RT-qPCR were consistent with those from RNA-Seq, thereby verifying the reliability of the transcriptomic results.

### The temporal mRNA expression profiles of DEGs following infection

**Figure 7:**
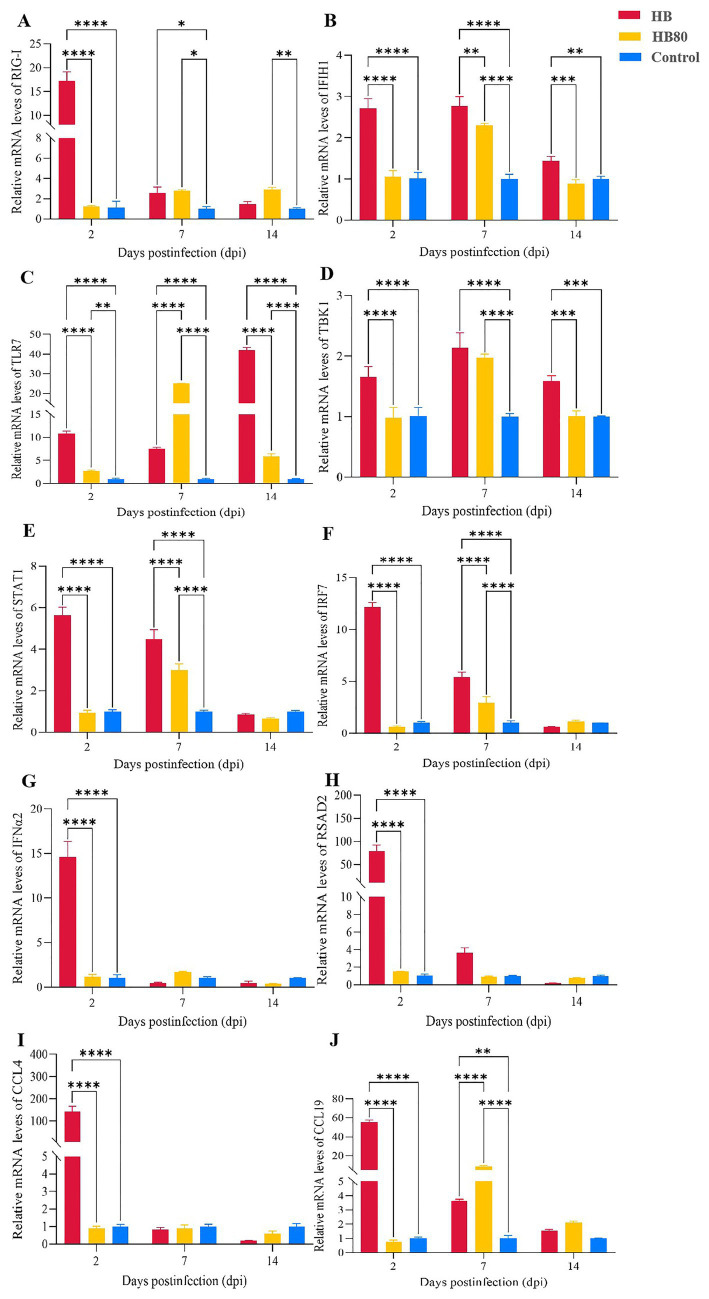
Hepatic mRNA expression of 10 core innate-immune genes at 2, 7, and 14 dpi. Relative expression levels of RIG-I **(A)**, IFIH1 **(B)**, TLR7 **(C)**, TBK1 **(D)**, STAT1 **(E)**, IRF7 **(F)**, RSAD2 **(G)**, IFN-α2 **(H)**, CCL4 **(I)**, and CCL19 **(J)** were quantified by RT-qPCR. Gene expression was normalized to β-actin and calculated using the 2^(−ΔΔCt)^ method. Data are presented as mean ± SD from three biological replicates (*n* = 3). Statistical significance is indicated as *p-value < 0.05, **p-value < 0.01, ***p-value < 0.001, ****p-value < 0.0001.

To further explore the expression patterns of DEGs at different time points after infection, mRNA expression levels of 10 key immune-related cytokines and genes were measured in livers at 2, 7, and 14 dpi (Figure 7). The expression dynamics of key immune-related genes revealed distinct host response patterns to the virulent HB strain vs. the attenuated HB80 vaccine strain.

For the PRRs, at 2 dpi, *RIG-I* expression surged 15.6-fold, markedly higher than in HB80-infected ducks. Similarly, *TLR7* expression increased sharply by 10.7-fold at 2 dpi in the HB group. A unique response was observed for TLR7 at 7 dpi, where the HB80 group exhibited a massive 25-fold induction, surpassing the HB group’s increase. This elevated TLR7 level in the HB80 group was later exceeded by the HB group at 14 dpi, which showed a sustained 41.9-fold upregulation. And *IFIH1* (*MDA5*) expression was strongly and early induced by the virulent HB strain, while the attenuated HB80 strain triggered a delayed and transient upregulation.

For the signaling molecules and transcription factors, *TBK1* showed consistent upregulation from 2 to 14 dpi in the HB group. The transcription factor IRF7 reached a peak early response at 2 dpi in the HB group (12.1-fold increase), far exceeding the response in the HB80 group. *STAT1* also displayed persistent elevation in HB-infected ducks, with a 5.63-fold increase at 2 dpi.

For the cytokine and antiviral effector responses, *IFN-α2* was strongly induced (14.0-fold) at 2 dpi in the HB group. Consequently, the antiviral effector *Rsad2* demonstrated extraordinary upregulation, reaching 77.4-fold in the HB group at the same time point, highlighting a potent ISG response. The transcription factor *IRF7* was most strongly upregulated by the virulent HB strain at 2 dpi, driving the subsequent interferon response, while the attenuated HB80 strain induced only a mild and transient increase.

For the chemokine responses, the HB strain caused an early and massive surge in pro-inflammatory chemokines at 2 dpi, with *CCL4* increasing 141.8-fold and *CCL19* increasing 55.1-fold. Notably, by 7 dpi, the expression pattern of CCL19 shifted, with the HB80 group showing a higher level (8.54-fold) than the HB group, suggesting a transition in immune cell recruitment.

### Protein expression levels of DEPs in livers following infection

**Figure 8:**
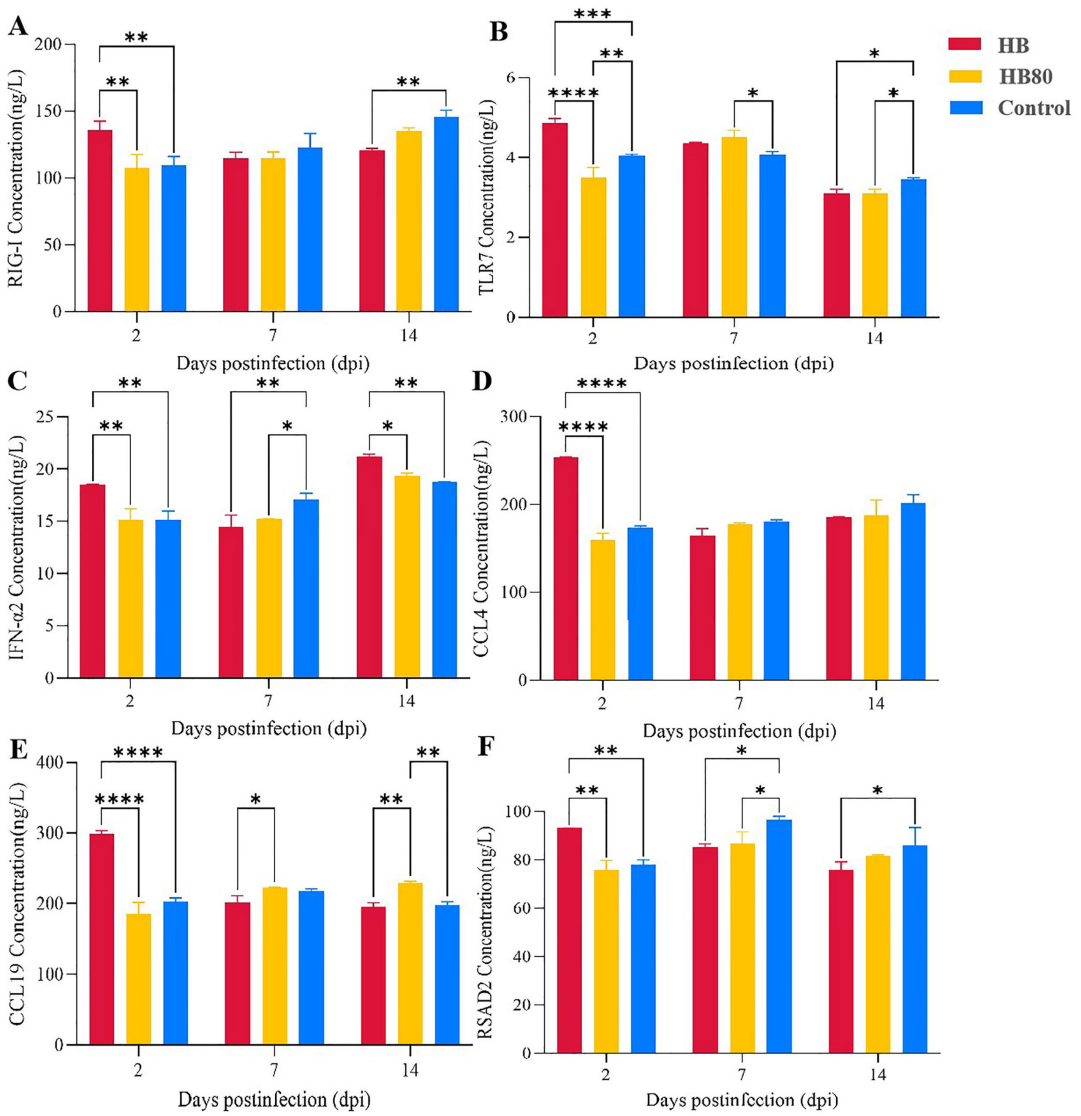
Hepatic protein expression levels of cytokines in ducklings post-infection. Protein levels of RIG-I **(A)**, TLR7 **(B)**, IFN-α2 **(C)**, CCL4 **(D)**, CCL4 **(E)**, and RSAD2 **(F)** were assessed by ELISA. Data are presented as mean ± SD from two biological replicates (*n* = 2). Statistical significance is indicated as follows: *p-value < 0.05, **p-value < 0.01, ***p-value < 0.001, ****p-value < 0.0001.

Protein expression levels by ELISA revealed distinct temporal expression patterns for key immune factors (Figure 8). Overall, the virulent HB strain generally induced stronger early protein-level upregulation at 2 dpi, whereas expression in both infection groups trended toward or below control levels at later time points.

PRRs and signaling: RIG-I and TLR7 proteins showed modest early increases in the HB group at 2 dpi (up to 1.39-fold), which subsequently declined. By 14 dpi, levels for both PRRs were slightly reduced across all groups.

Cytokine and antiviral effector: IFN-α2 and the antiviral protein RSAD2 followed a similar pattern, with peak expression in the HB group at 2 dpi (up to 1.23-fold) before decreasing.

Chemokines: The chemokines CCL4 and CCL19 exhibited the most pronounced changes. Both were markedly upregulated by the HB strain at 2 dpi (peaking at 1.61-fold for CCL19). Notably, CCL19 expression dynamics diverged at later stages, with the HB80-immunized group sustaining a higher level than the HB-infected group at 7 and 14 dpi.

## Discussion

Duck hepatitis A virus remains the predominant causative agent of viral hepatitis in ducklings, leading to severe economic losses worldwide. Following the widespread application of attenuated live vaccines against DHAV-1, DHAV-3 has emerged as predominant serotype responsible for outbreaks, highlighting the need for a deeper understanding of its pathogenesis and vaccine-induced immunity (Ou et al., 2022; Wen et al., 2018). This study elucidates the distinct host responses to virulent and attenuated DHAV-3 strains through integrated viral kinetics, transcriptomic, and protein-level analyses.

Consistent with previous reports on virulent strains, the HB strain exhibited rapid replication, peaking at 2 days post-infection (dpi) with a load of 10^4.7^ copies/μg cDNA before gradually declining (Niu et al., 2019; Shawki et al., 2024). In contrast, the attenuated HB80 vaccine strain reached a peak titer approximately 1,900-fold lower, demonstrating restricted replication. This pivotal peak at 2 dpi was selected for comparative transcriptomic analysis to capture the differential host responses at the height of viral activity.

While previous transcriptomic studies have advanced our understanding of DHAV-3 pathogenesis (Cao et al., 2020; Liang et al., 2021, 2022; Zhang et al., 2018a,b), including the roles of metabolic disruption and immune hyperactivation (Zhang et al., 2018a,b) and more recently, PLAC8 and complement-coagulation axis dysregulation at single-cell resolution (Zhang et al., 2025), the mechanistic distinctions in host responses to virulent vs. attenuated strains, particularly in immune regulation, remain poorly characterized. This study systematically addresses this gap by comparing transcriptomic alterations induced by the virulent HB strain and the attenuated HB80 vaccine strain.

Innate immunity is the first line of defense against DHAV. Prior proteomic work showed enrichment of RLR, TLR, and NLR pathways during DHAV-3 infection (Liang et al., 2020; Zhang et al., 2018a,b). Consistent with this, we found the virulent HB strain induced markedly more pronounced enrichment of multiple immune pathways than HB80, including these PRR pathways, cytokine-cytokine receptor interactions, and chemokine signaling.

Critical PRRs like TLR7, MDA5, and RIG-I are essential for sensing RNA viruses and initiating immune cascades (Landström, 2010; del Toro Duany et al., 2015). Our data reveal that early HB infection (2 dpi) synergistically hyperactivates the TBK1-IRF7 signaling axis, driven by coordinated engagement of RIG-I/MDA5 and TLR7. This led to a profound disruption of immune homeostasis. TBK1 and IRF7 mRNA were upregulated approximately 9.8-fold and 12.3-fold in HB-infected ducks, far exceeding the 4.1-fold and 5.2-fold inductions in the HB80 group. This IRF7 activation drove a robust 14.0-fold upregulation of IFN-α2 and subsequent overexpression of ISGs. Notably, the antiviral effector RSAD2 showed a dramatic 77.4-fold mRNA upregulation with concordant protein elevation in HB-infected hosts. Given the sequence variation in the viral 2C protein between the HB and HB80 strains (Fu et al., 2024), and evidence that RSAD2 can interact with viral 2C proteins to inhibit replication (Hou et al., 2024), we hypothesize that an RSAD2-2C interaction may modulate interferon signaling and influence viral pathogenicity, a premise warranting further study. This potent interferon storm in HB infection resulted from dual-pathway synergy: TLR7 (10.7-fold mRNA upregulation) and RIG-I/MDA5 signals converged excessively on the TBK1-IRF7 axis, rather than the canonical MyD88-NF-κB pathway, leading to amplified IFN-I production. This hyperactivated, multi-pathway-amplified IFN-I cascade is a central pathogenic driver of the hepatic immunopathology underlying lethal hepatitis.

Chemokines orchestrate immune cell recruitment. KEGG analysis revealed significantly upregulated cytokine-receptor networks. At 2 dpi, HB infection caused dramatically higher upregulation of key chemokines CCL4 (141.8-fold) and CCL19 (55.1-fold) compared to HB80, which likely triggers excessive monocyte/macrophage recruitment and exacerbates inflammatory damage. Intriguingly, the HB80 group demonstrated a unique capacity for immunomodulation, with its CCL19 expression surpassing HB-infected levels by 7 dpi. This shift may facilitate lymphocyte-specific recruitment and memory formation, underscoring its vaccine candidacy.

In contrast to the pathological hyperinflammation induced by HB, the attenuated HB80 strain established a more balanced and temporally modulated immune profile. While it activated interferon responses, the upregulation of key mediators (TBK1, IRF7, IFN-*α*2, ISGs) was significantly lower. Crucially, the peak expression of IRF7 and STAT1 was delayed until 7 dpi, preventing the acute interferon storm. Furthermore, KEGG analysis indicated that HB80 infection activates the PI3K-Akt signaling pathway and antigen processing and presentation cascades, suggesting a strategic shift toward adaptive immunity and T-cell responses.

**Figure 9:**
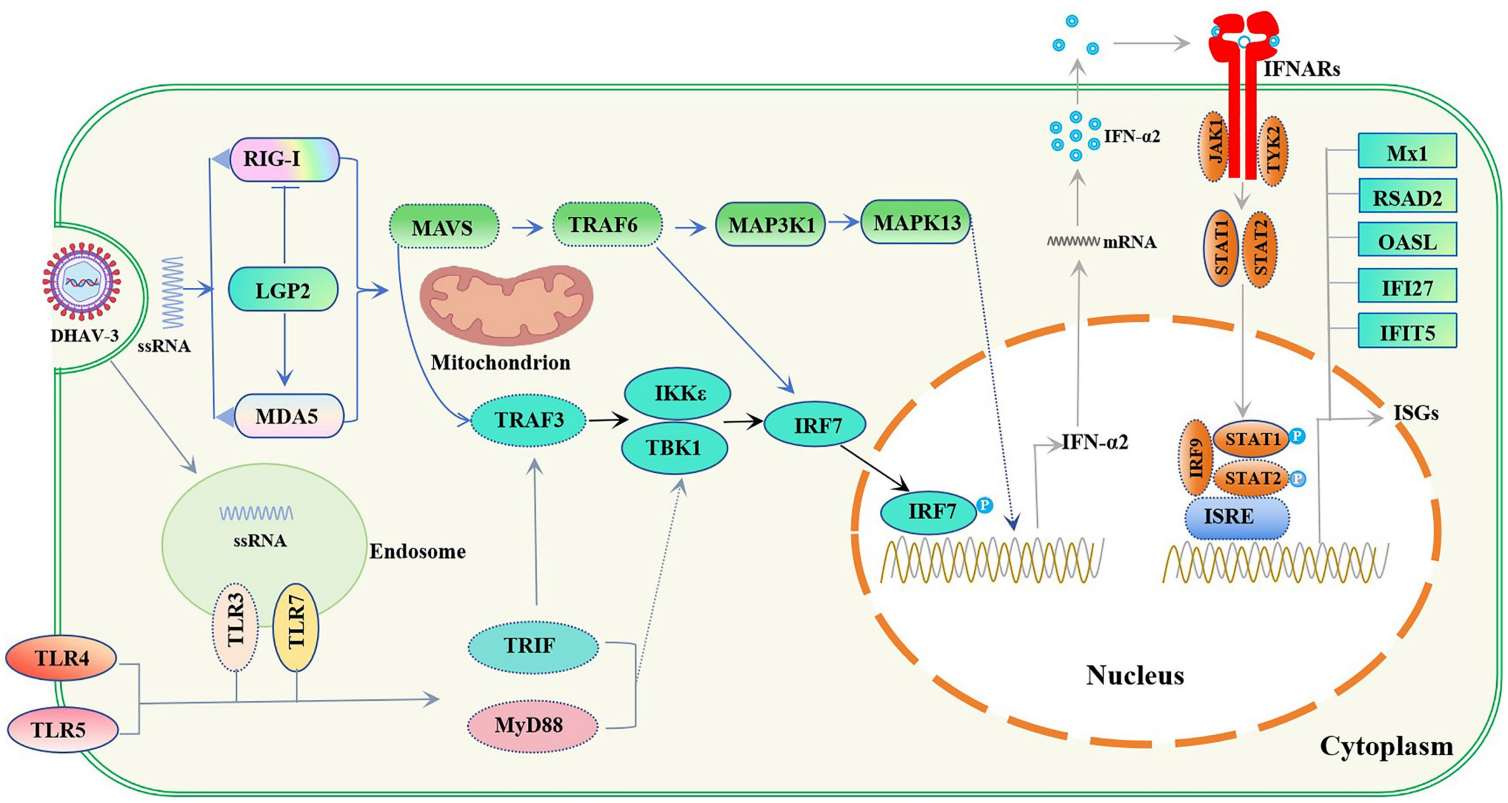
Schematic of the innate immune response induced by DHAV-3 in the duckling liver. The schematic illustrates the signaling pathways and key elements identified. Components represented by solid boxes correspond to DEGs detected in the transcriptome analysis of DHAV-3-infected livers, whereas the remaining undetected DEPs are shown in the dotted boxes.

This study delineates two distinct host interaction strategies: the virulent HB strain causes pathological hyperinflammation via a synergistic, TBK1-IRF7-mediated interferon storm, while the attenuated HB80 vaccine strain induces a balanced, delayed, and adaptive immunity-skewed response that ensures protection without significant immunopathology. These findings provide a mechanistic foundation for understanding DHAV-3 pathogenesis and vaccine-induced protection (Figure 9).

## Conclusion

Our data identify the TBK1-IRF7-IFN-α axis and parallel TLR7/RIG-I/MDA5 signaling as the key differentiating pathways between virulent HB and vaccine HB80 infections. Virulent challenge drove an acute, high-magnitude interferon storm accompanied by 140-fold CCL4 and 77-fold RSAD2 induction that precipitated hepatic injury. However, HB80 elicited only modest, delayed innate activation while simultaneously up-regulating PI3K-Akt and antigen-presentation modules consistent with adaptive priming. Compared with previous transcriptomic studies that profiled only virulent DHAV-3, our side-by-side comparison reveals the qualitative and quantitative immune signatures that distinguish protection from pathology, providing a mechanistic framework for next-generation live-attenuated or subunit vaccines against DHAV-3.

## Data Availability

The datasets presented in this study can be found in online repositories. The names of the repository/repositories and accession number(s) can be found in the article/Supplementary material.

## Footnotes

1
http://string-db.org/
