# Correction: Comparative analysis of ducks liver gene expressions infected with virulent or attenuated DHAV-3 reveals divergent host responses to viruses of different virulence

**DOI:** 10.3389/fmicb.2026.1853750

**Published:** 2026-04-21

**Authors:** Frontiers Production Office

**Affiliations:** Frontiers Media SA, Lausanne, Switzerland

**Keywords:** duck hepatitis A virus type 3, IFN-I, immunity, transcriptome, virulence

A correction has been made to the section Abstract: **Discussion**:

“These findings delineate divergent host transcriptomic responses to virulent vs. attenuated DHAV-3 and highlight IFN-I signaling as a central axis in antiviral immunity.”

The original version of this article has been updated.

